# Retraction: A novel triple repeat mutant tau transgenic model that mimics aspects of pick’s disease and fronto-temporal tauopathies

**DOI:** 10.1371/journal.pone.0325329

**Published:** 2025-05-28

**Authors:** The PLOS One Editors

Following the publication of this article [1], concerns were raised regarding results presented in Figs 2, 7, 9, and 11. Specifically,

- There appear to be similarities between lanes within numerous panels presented in Fig 2A.
- There appear to be irregularities in the background of the Fig 2A Actin panel.
- Similarities were noted between multiple regions within the following panels:
  - Fig 9A Non-tg DG cell
  - Fig 11A 3R Tau 3R Tau (Line 2)
- The Fig 7D MAP2 Non-tg and MAP2 3R Tau tg 6-8 m panels appear to partially overlap.
- The Fig 9A Non-tg Axon panel in this article [1] appears similar to the Fig 9E panel of [2] despite representing different experimental conditions.

Regarding the similarities within the western blot panels in Fig 2A, the corresponding author stated that this figure was included for illustration purposes only. The corresponding author provided some underlying image data, but these data were insufficient to resolve the concerns with the published figure and PLOS remains concerned that the areas in the panels appear more similar than would be expected from independent results.

The corresponding author stated that errors were made during the preparation of Figs 7 and 9, such that the Fig 7D MAP2 Non-tg panel and the Fig 9A Non-tg Axon panel are incorrect.

Regarding the repetitive elements observed in Figs 9 and 11, the corresponding author stated that “microduplications” may have occurred during the processing of the image data. They provided images underlying Figs 9 and 11. Editorial assessment of these underlying data raised further concerns about the reliability and integrity of these data.

In light of the above concerns that call into question the reliability and integrity of the published results, the *PLOS One* Editors retract this article.

BS, EM, and CRO did not agree with the retraction and stand by the article’s findings. ER, KU, MM, CP, AA, AB, and MTM either did not respond directly or could not be reached.

The Fig 9A Non-tg Axon panel reports material from [2], published in 2013 by Nuber et al, which is not available under a CC BY 4.0 license and is therefore excluded from the article’s [1] license.
